# Vancomycin levels for Bayesian dose-optimization in critical care: a prospective cohort study

**DOI:** 10.3389/fmed.2025.1575224

**Published:** 2025-07-22

**Authors:** Natalia Dreyse, Nicole Salazar, Jose M. Munita, Jordi Rello, René López

**Affiliations:** ^1^Departamento de Paciente Crítico, Clínica Alemana de Santiago, Santiago, Chile; ^2^Departamento de Farmacia, Clínica Alemana de Santiago, Santiago, Chile; ^3^Genomics & Resistant Microbes Group (GeRM), Instituto de Ciencias e Innovación en Medicina (ICIM), Facultad de Medicina, Clínica Alemana, Universidad del Desarrollo, Santiago, Chile; ^4^CRIPS Research Group-Vall d’Hebrón Institute Research, Barcelona, Spain; ^5^Formation, Recherche, Assessment (FOREVA), CHU Nîmes, Nîmes, France; ^6^Centro Investigación Biomédica en Red (CIBERES), Instituto Salud Carlos III, Madrid, Spain; ^7^Grupo Intensivo, Instituto de Ciencias e Innovación en Medicina (ICIM), Facultad de Medicina, Clínica Alemana, Universidad del Desarrollo, Santiago, Chile

**Keywords:** pharmacokinetics, area under curve/minimum inhibitory concentration, intensive care unit, glycopeptides, antibiotics, sepsis

## Abstract

**Background:**

Vancomycin dosing in critically ill patients typically requires monitoring the area under the concentration-time curve/minimum inhibitory concentration (AUC/MIC), often using at least two vancomycin levels (VLs). However, the optimal number of VLs needed for accurate AUC/MIC estimation in this population remains uncertain. This study aimed to determine the minimum number of VLs required to accurately estimate the AUC/MIC in critically ill patients treated with intermittent infusion of vancomycin.

**Methods:**

A prospective cohort study was conducted in critically ill patients, where VLs were obtained at peak, beta, and trough phases. Five AUC estimates were derived using PrecisePK^™^, a Bayesian software: AUC-1 [peak, beta (2 h after the end infusion), trough], AUC-2 (beta, trough), AUC-3 (peak, trough), AUC-4 (trough), and AUC-5 (only Bayesian prior, without VL). These estimates were compared for accuracy and bias (mean ± SEM) against the reference AUC calculated via the trapezoidal model (AUC_Ref_).

**Results:**

We enrolled 36 adult patients with age of 65 (52–77) years, moderate severity [APACHE II 10 (5–14) and SOFA 5 (4–6)], 6 of them in ECMO and 4 in renal replacement therapy. A total of 108 blood samples for VL were analyzed. The AUC-3 (0.976 ± 0.012) showed greater accuracy compared to AUC-4 (1.072 ± 0.032, *p* = 0.042) and AUC-5 (1.150 ± 0.071, p = 0.042). AUC-3 also demonstrated lower bias (0.053 ± 0.009) than AUC-4 (0.134 ± 0.026, *p* = 0.036) and AUC-5 (0.270 ± 0.060, *p* = 0.003). Bland–Altman analysis indicated better agreement between AUC-3 and AUC-2 with AUC_Ref_.

**Conclusion:**

Bayesian software using two vancomycin levels provides a more accurate and less biased AUC/MIC estimation in critically ill patients.

## 1 Introduction

Vancomycin, a glycopeptide antibiotic, is widely used to treat Gram-positive bacterial infections, particularly in serious Methicillin-resistant *Staphylococcus aureus* (MRSA) infections (1). However, its narrow therapeutic window presents challenges: excessive concentrations can lead to nephrotoxicity, while subtherapeutic levels may result in treatment failure (2–5). In fact, the rate of nephrotoxicity reported in patients treated with intermittent infusion of vancomycin up to 19% (1).

**Figure 1:**
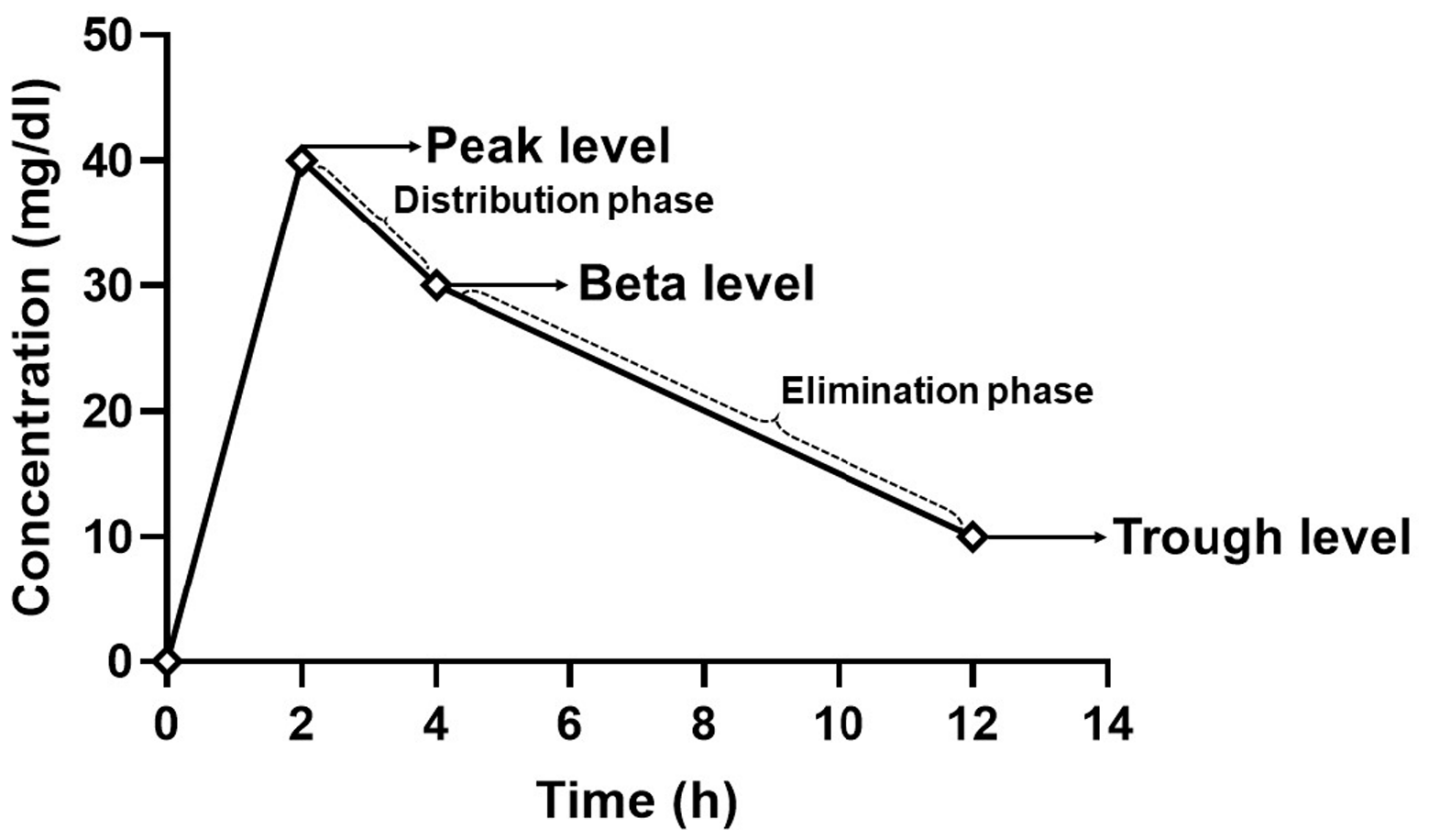
Timing of vancomycin levels in a schematic representation, where peak level was measured 20 min post-infusion completion; beta level was measured 2 h post-infusion completion; and trough level was measured 1 h before the next scheduled vancomycin dose. The distribution phase reflects the rapid decline due to drug distribution into tissues, followed by the beta level, marking the beginning of the elimination phase, where the drug is excreted. The trough level denotes the lowest concentration before the next dose, critical for therapeutic monitoring.

Vancomycin follows a two-compartment pharmacokinetic model, characterized by an initial tissue distribution phase lasting approximately 2 h post-infusion, followed by a prolonged elimination phase (Figure 1) (6, 7). This complexity, coupled with its toxicity potential, necessitates careful therapeutic monitoring to optimize efficacy while minimizing adverse effects (6). The area under the concentration-time curve to minimum inhibitory concentration (AUC/MIC) is the key pharmacokinetic/pharmacodynamic (PK/PD) parameter associated with vancomycin’s efficacy. An AUC/MIC target range of 400–600 h^−1^ is recommended (1, 6, 7). Traditionally, AUC/MIC calculations using the trapezoidal method required at least three plasma levels, making this approach cumbersome in clinical practice. Consequently, a trough level of 15–20 mg/L has been proposed as a surrogate marker in patients with normal renal function (8–11). Trough-based monitoring is a conventional approach for guiding vancomycin dosing by measuring the lowest drug concentration in plasma, typically just before the next dose. This method has shown limited correlation with the pharmacodynamic target (AUC/MIC ≥400), leading to increased risk of nephrotoxicity and reduced precision in individualized dosing (1).

The introduction of Bayesian pharmacokinetic modeling software has simplified AUC/MIC estimation by integrating variables such as patient demographics, renal function, and vancomycin plasma levels (12–19). This approach enables rapid and accessible PK/PD predictions. However, critically ill patients pose unique pharmacokinetic challenges, including altered renal function and hemodynamic instability, which complicate dose optimization (20–22). Additionally, these patients are at higher risk of vancomycin-associated nephrotoxicity due to factors like sepsis, hypovolemia, and concomitant nephrotoxic therapies (2, 23, 24).

While Bayesian software has streamlined AUC/MIC estimation, the optimal number of vancomycin plasma levels required for accurate calculations in critically ill patients remains unclear (25). Current guidelines recommend two plasma levels (6, 9), whereas some Bayesian software manufacturers suggest only one (13, 14, 26). This study aims to address this uncertainty by determining the minimum number of plasma levels needed for accurate and reliable AUC/MIC estimation in critically ill patients treated with intermittent infusion of vancomycin. We hypothesize that at least two plasma levels are necessary to achieve precise AUC/MIC estimation in this population.

## 2 Patients and methods

### 2.1 Study design and patient selection

This prospective observational study consecutively recruited critically ill patients treated with vancomycin between October 2021 and June 2022 to evaluate the number of vancomycin levels (VLs) required for accurate AUC/MIC estimation. Five AUC/MIC estimation methods, incorporating 0, 1, 2, or 3 VLs, were compared to a standard reference method. Patients were followed until hospital discharge.

Eligible participants were adults aged 18 years or older admitted to the intensive care unit (ICU). Inclusion criteria included patients receiving vancomycin as targeted therapy for a documented or suspected Gram-positive infection. In addition, patients were required to have three vancomycin plasma concentrations obtained during the treatment period.

Patients were excluded from the analysis if they did not have at least three vancomycin plasma concentrations obtained during the treatment period. This criterion was established to ensure sufficient pharmacokinetic data for accurate AUC estimation and model reliability. The absence of a minimum of three levels prevented robust Bayesian analysis and therefore precluded their inclusion in the final cohort.

This study was conducted at our ICU in accordance with the ethical standards of the Declaration of Helsinki (1975, revised 2013) and institutional or regional guidelines. Approval was obtained from the local Ethics Board (IRB00011516, Approval Number 2012/53). Given its observational nature, the requirement for informed consent was waived by the committee.

### 2.2 Vancomycin administration

Vancomycin is reconstituted with sterile water for injection to a concentration of 50 mg/mL and then diluted in a compatible intravenous fluid, such as 0.9% sodium chloride (normal saline) or 5% dextrose (D5W) to achieve a final concentration of 10 mg/mL, for all patients included in patients requiring fluid restriction (27).

Vancomycin intermittent infusion administration for central venous catheter is infused over at least 60 min for dose 1 g or less, while higher doses require 120 min, to minimize infusion-related reactions the infusion rate should not exceed 10 mg/min, which may require an infusion time of 1 to 2 h in order to avoid surpassing the rate associated with the highest incidence of adverse events (1).

### 2.3 Vancomycin plasma level measurements

To determine the area under the concentration-time curve to minimum inhibitory concentration (AUC/MIC), three distinct vancomycin plasma levels were measured using kinetic interaction of microparticles in a solution (KIMS) COBAS, Roche^™^ (Figure 1):

Peak level: Measured 20 min after infusion completion.
Beta level: Measured 2 h after infusion completion.
Trough level: Measured 1 h before the next scheduled dose.

These levels were used to calculate the pharmacokinetic profile of vancomycin for each patient.

The measurements were taken based on protocol number 6630 “Protocolo de uso de vancomicina en pacientes hospitalizados adultos.”

### 2.4 Calculation of referential AUC

The referential AUC (AUC_Ref_) was calculated using the trapezoidal rule (28), incorporating the peak, beta, and trough plasma levels. This method provided the standard against which other AUC estimates were compared, representing the total exposure to vancomycin over time. Then, AUC was divided by MIC and when MIC was not available, we assumed a MIC = 1 mg/L. A MIC value of 1 mg/L is used as a reference for vancomycin because the Infectious Diseases Society of America (IDSA) guideline states that under most circumstances of empiric dosing, the vancomycin MIC should be assumed to be 1 mg/L (1).

### 2.5 Bayesian pharmacokinetics software

The commercially available Bayesian software PrecisePK^™^ was used for dose optimization and AUC/MIC estimation. Five approaches were evaluated:

AUC-1: Incorporating all three levels (peak, beta, and trough).
AUC-2: Incorporating beta and trough levels.
AUC-3: Incorporating peak and trough levels.
AUC-4: Using the trough level alone.
AUC-5: Based solely on PrecisePK^™^ Bayesian prior assumptions without plasma level data.

This comprehensive approach enabled the evaluation of vancomycin dosing strategies across various clinical scenarios in critically ill patients.

PrecisePK^™^ is a cloud-based clinical decision support tool that uses Bayesian forecasting to estimate individualized pharmacokinetic (PK) parameters and predict vancomycin exposure (AUC) in real time. It incorporates validated population PK models and integrates patient-specific covariates (e.g., weight, age, renal function) and measured vancomycin levels to refine predictions.

Compared to other Bayesian platforms such as InsightRx^™^ or DoseMeRx^™^, PrecisePK^™^ offers an intuitive interface, rapid cloud-based computation, and customizable institutional protocols. One notable distinction is its FDA 510(k) clearance, which supports its clinical integration and regulatory compliance (29). Additionally, PrecisePK has been externally validated in critically ill populations, demonstrating accurate AUC estimation and clinical utility (10, 30).

### 2.6 Statistical analysis

Due to the exploratory nature of this study we do not provide a sample size estimation.

Statistical analyses were conducted in three phases to ensure robust evaluation of the data:

Comparison of AUC_Ref_/MIC with AUC_(estimated)_/MIC.

Differences between these values were assessed to determine statistical significance.

Correlation analysis.

The linear relationship between AUC_Ref_/MIC and AUC_(estimated)_/MIC was evaluated using Pearson’s correlation coefficient. Bland–Altman analysis was also performed to examine limits of agreement (LOA) between these measures, providing insight into their concordance.

Accuracy and bias assessment.

Accuracy: Defined as the ratio of AUC_(estimated)_/MIC to AUC_Ref_/MIC, offering a comparative measure against the reference standard.

Bias: Calculated as the absolute difference between AUC_(estimated)_/MIC and AUC_Ref_/MIC, normalized to AUC_Ref_/MIC.

To compare overall group differences, the Kruskal–Wallis *H* test was used, followed by Mann–Whitney *U* tests for subgroup analyses. Statistical significance was set at *p* < 0.05. All analyses were performed using SPSS software (version 20.0, SPSS Inc., Chicago, IL, United States).

## 3 Results

**Figure 2:**
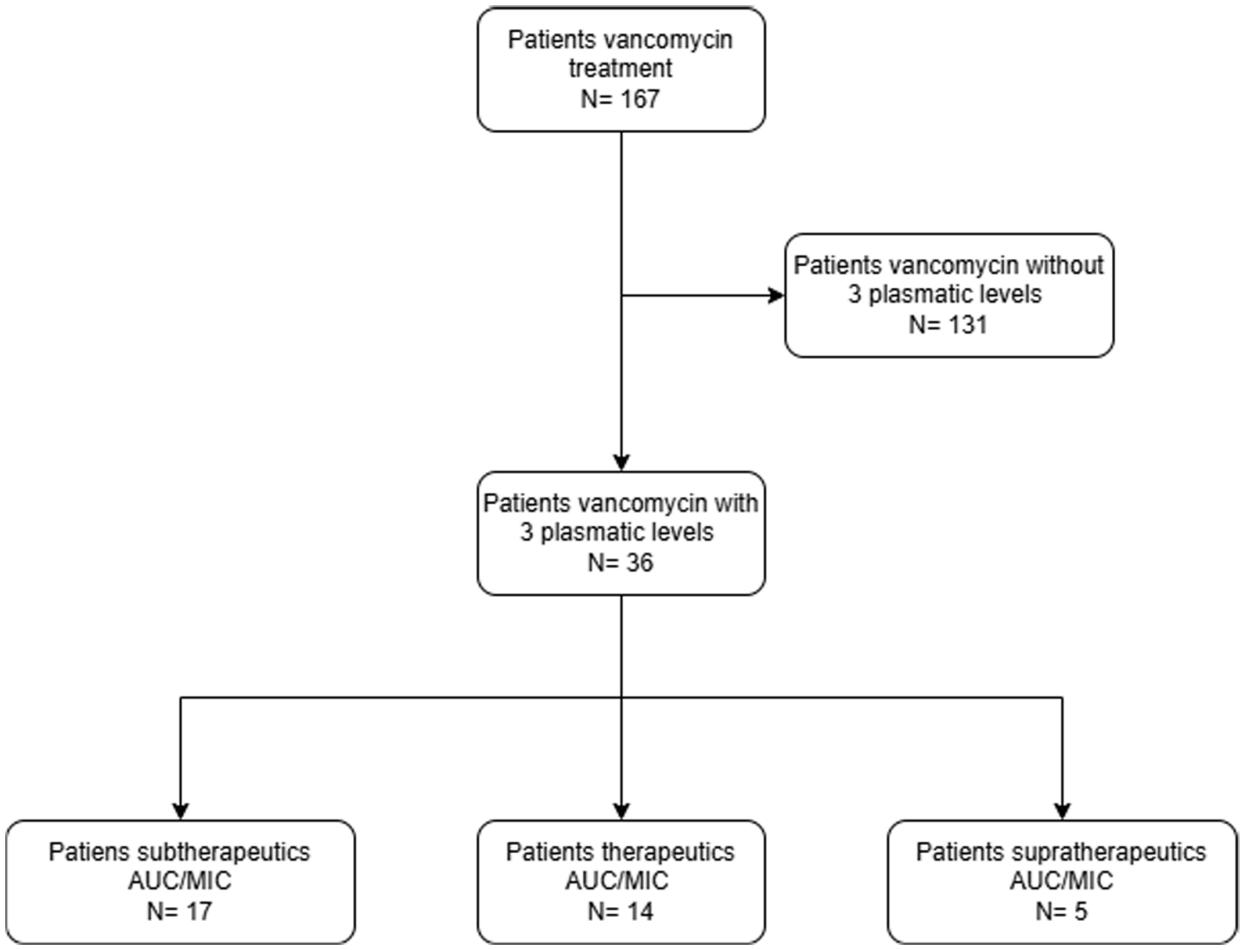
Flowchart illustrating the selection process of patients included in the vancomycin pharmacokinetic analysis. Out of 167 patients who received vancomycin, 131 were excluded because they did not have three plasma level measurements required for AUC/MIC calculation. The remaining 36 patients with complete pharmacokinetic data were included in the analysis and categorized according to their AUC/MIC ratio into subtherapeutic (*n* = 17), therapeutic (*n* = 14), and supratherapeutic (*n* = 5) groups.

During the study period, 167 adult patients received vancomycin in the ICU, of whom only 36 met the inclusion criteria (see flowchart in Figure 2) with 108 blood samples collected for VL. Among the 36 enrolled patients, 26 (72.2%) were male. Two-thirds required invasive mechanical ventilation, and 4 (16.7%) were connected to extracorporeal membrane oxygenation (ECMO). Most patients had preserved renal function, although 8.3% required continuous renal replacement therapy (CRRT). The overall hospital mortality rate was 17% (Table 1).

**Table 1:** Characterization of patients.

Variable	Values
*N*	36
Demography and anthropometry	
Male, %	72.2
Age, year	65 (52–77)
Weight, kg	79 (65–90)
Height, cm	170 (161–176)
BMI, kg/m^2^	28 (24–31)
Severity	
APACHE II, score	10 (5–14)
SOFA, score	5 (4–6)
Mechanical ventilation, %	66.7
ECMO, %	16.7
Serum creatinine, mg/dL	0.75 (0.58–0.98)
GFR, MDRD mL/min	92 (76–131)
GFR, CG mL/min	108 (86–136)
GFR, CG mL/min/1.73 m	90 (77–118)
CRRT, %	8.3
Comorbidities	
Chronic comorbidities, %	94.4
Hypertension, %	47.1
Diabetes, %	29.4
Dyslipidemia, %	8.8
Obesity, %	29.4
COPD, %	11.7
Asthma, %	5.8
ESKD, %	2.9
Immunological disease, %	20.6
Active cancer, %	38.2
Outcomes	
ICU LOS, days	19 (6–71)
Hospital LOS, days	38 (20–89)
Hospital mortality, %	16.6

BMI, body mass index; APACHE II, acute physiology and chronic health evaluation; SOFA, sequential organ failure assessment; ECMO, extracorporeal membrane oxygenation; GFR, glomerular filtration rate; MDRS, modification of diet in renal disease; CG, Cockroft-Gault; CRRT, continuous renal replacement therapy; COPD, chronic obstructive pulmonary disease; ESKD, end-stage kidney disease; ICU, intensive care unit; and LOS, length of stay.

We identified 27 patients with positive culture while 9 patients were treated with vancomycin as part of an empiric therapy. For patients in whom a MIC was not available, a MIC = 1 mg/L was assumed. Bloodstream infections were the most common (17 patients, 47.2%), followed by respiratory infections. The predominant pathogens were *Staphylococcus epidermidis* (33.3%) and cursive faecalis (13.9%) (Table 2). At study enrollment, the majority (72.2%) of patients received a vancomycin dose of 1,000 mg every 12 h (Q12h) with a standard infusion duration of 1 h (Table 3).

**Table 2:** Description of infection source and bloodstream associated bacterial pathogens.

Variable	Values %
Infection source	
Abdominal	8.3
Bacteremia	33.3
Central nervous system	11.1
Respiratory	19.4
Skin and soft tissues	11.1
Urinary tract	16.7
Bloodstream	
Yes	47.2
Bacteria	
*Enterococcus faecalis*	13.9
*Enterococcus faecium*	2.8
*Staphylococcus aureus*	8.3
*Staphylococcus capitis*	2.8
*Staphylococcus epidermidis*	33.3
*Staphylococcus haemolyticus*	5.5
*Staphylococcus hominis*	2.8
*Streptococcus anginosus*	2.8
*Streptococcus intermedius*	2.8
No microorganisms detected	25.0
MIC	
0.5 mg/L	13.9
1 mg/L	58.3
2 mg/L	5.6
No data	22.2

**Table 3:** Therapy characteristics.

Variable		Values *N* (%)
Vancomycin dose, mg	500	4 (11.1)
	750	4 (11.1)
	1,000	26 (72.2)
	1,500	1 (2.7)
	2,000	1 (2.7)
Vancomycin dose interval, hour	Q8	7 (19.4)
	Q12	28 (77.7)
	Q24	1 (2.7)
Vancomycin infusion duration, hour	1	25 (69.4)
	2	11 (30.5)

### 3.1 Pharmacokinetic analysis

**Figure 3:**
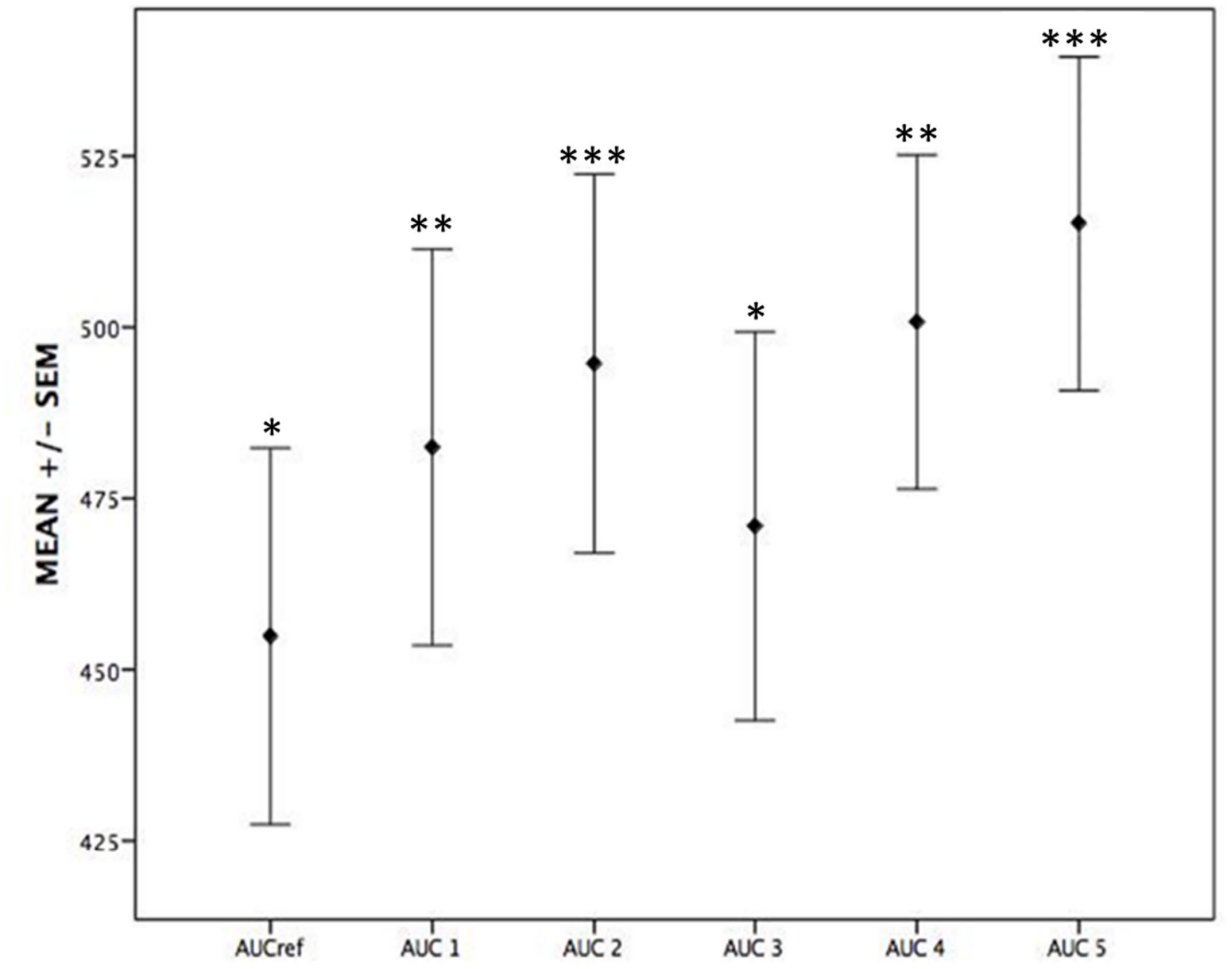
Mean values ± standard error of the mean (SEM) for each experimental condition (AUCref, AUC1–AUC5). Statistical comparisons were performed against the reference group (AUCref), with symbols indicating significant differences: ^*^p < 0.05, ^**^p < 0.01, and ^***^p < 0.001.

The median area under the concentration-time curve (AUC) calculated using the linear-log trapezoidal rule (AUC_Ref_) was 435 (343–549) mg h/L, while the median AUC estimated using three vancomycin plasma levels with Bayesian software was 473 (367–535) mg h/L (Table 4). Notably, AUC_Ref_ values were consistently lower than all AUC estimation methods (Figure 3).

**Table 4:** Vancomycin AUC and plasmatic concentration values, where AUC_24_ is area under the curve in 24 h.

Variable		Median (*p*25–*p*75)	95% CI
Plasmatic level vancomycin, mg/dL	Trough	11 (9–16)	(7, 14)
	Beta	20 (16–25)	(15, 24)
	Peak	28 (23–39)	(19, 36)
AUC_24_, mg*h/L	AUC_Ref_: Linear-long trapezoidal rule	435 (343–549)	(329, 540)
	AUC-1: Bayesian program three levels	473 (367–535)	(386, 559)
	AUC-2: Bayesian program trough and beta level	469 (387–568)	(376, 561)
	AUC-3: Bayesian program trough and peak level	464 (366–539)	(375, 552)
	AUC-4: Bayesian program only trough level	495 (415–592)	(404, 585)
	AUC-5: Bayesian priori model	501 (419–591)	(413, 588)

**Figure 4:**
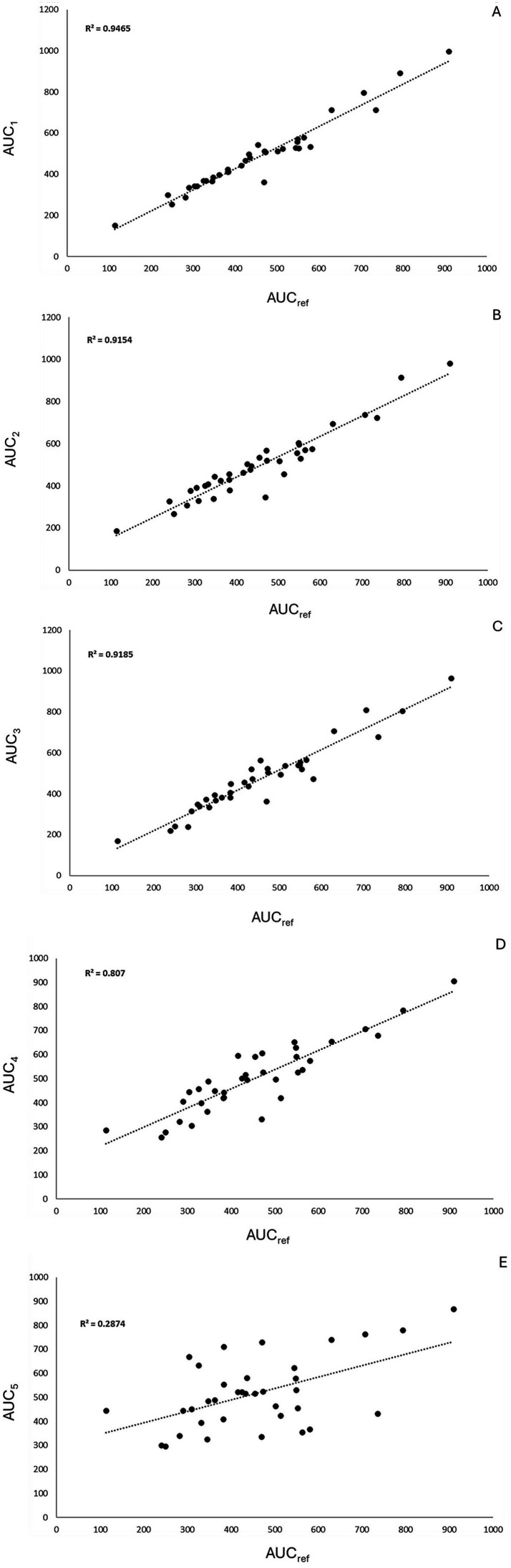
Correlation between observed and predicted values using different pharmacokinetic models. Graphics **A–E** show scatter plots comparing observed versus predicted drug concentrations for five different modeling approaches. Each graphic reports the coefficient of determination (*R*^2^), Pearson correlation coefficient (*r*), and significance level (*p* < 0.001). The dashed line represents the line of identity. Model **A** shows the strongest correlation (*R*^2^ = 0.946, *r* = 0.973), while model **E** shows the weakest performance (*R*^2^ = 0.287, *r* = 0.536), indicating variability in predictive accuracy among the tested models.

**Figure 5:**
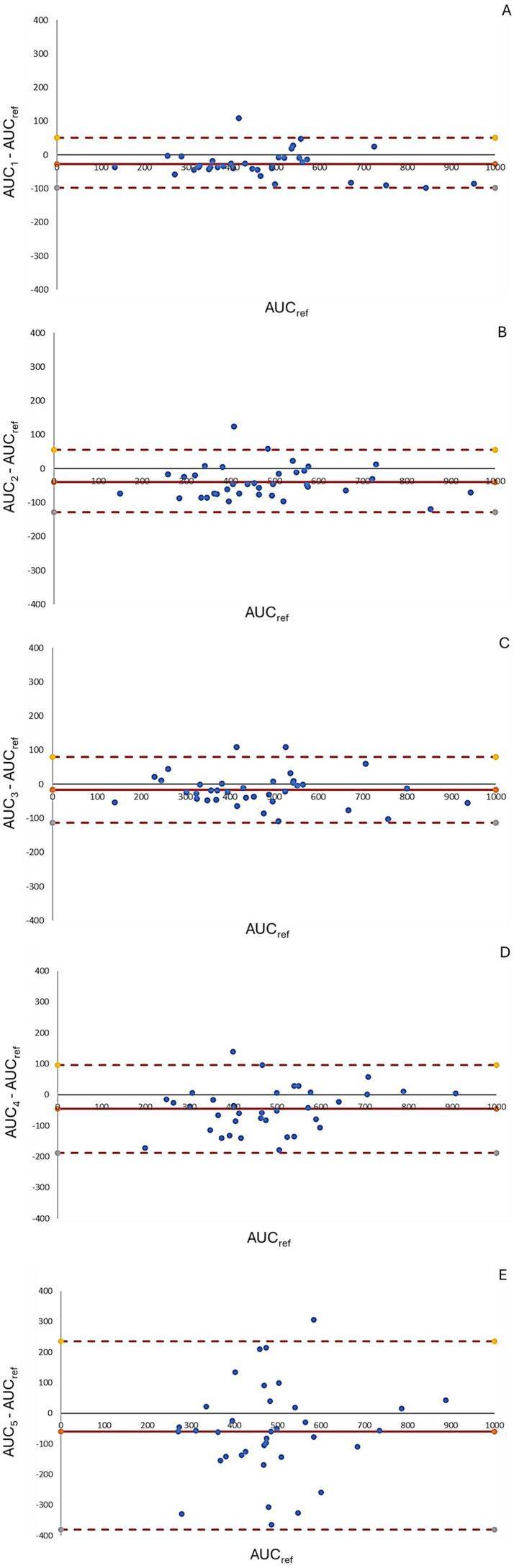
Bland–Altman plots comparing AUC estimations across different pharmacokinetic models. Graphics **A–E** depict Bland–Altman plots assessing the agreement between predicted and reference AUC values for five different pharmacokinetic models. The y-axis represents the percentage difference relative to the reference AUC, calculated as [(AUC_(estimated)_ − AUC_ref/_AUC_ref_)]. The solid red line indicates the mean bias, while the dashed red lines represent the 95% limits of agreement. Narrower limits and points closer to zero indicate better agreement. Graphic **A** shows the best agreement with minimal bias and tight limits, while graphic **E** displays greater variability and wider limits, indicating poorer concordance.

**Figure 6:**
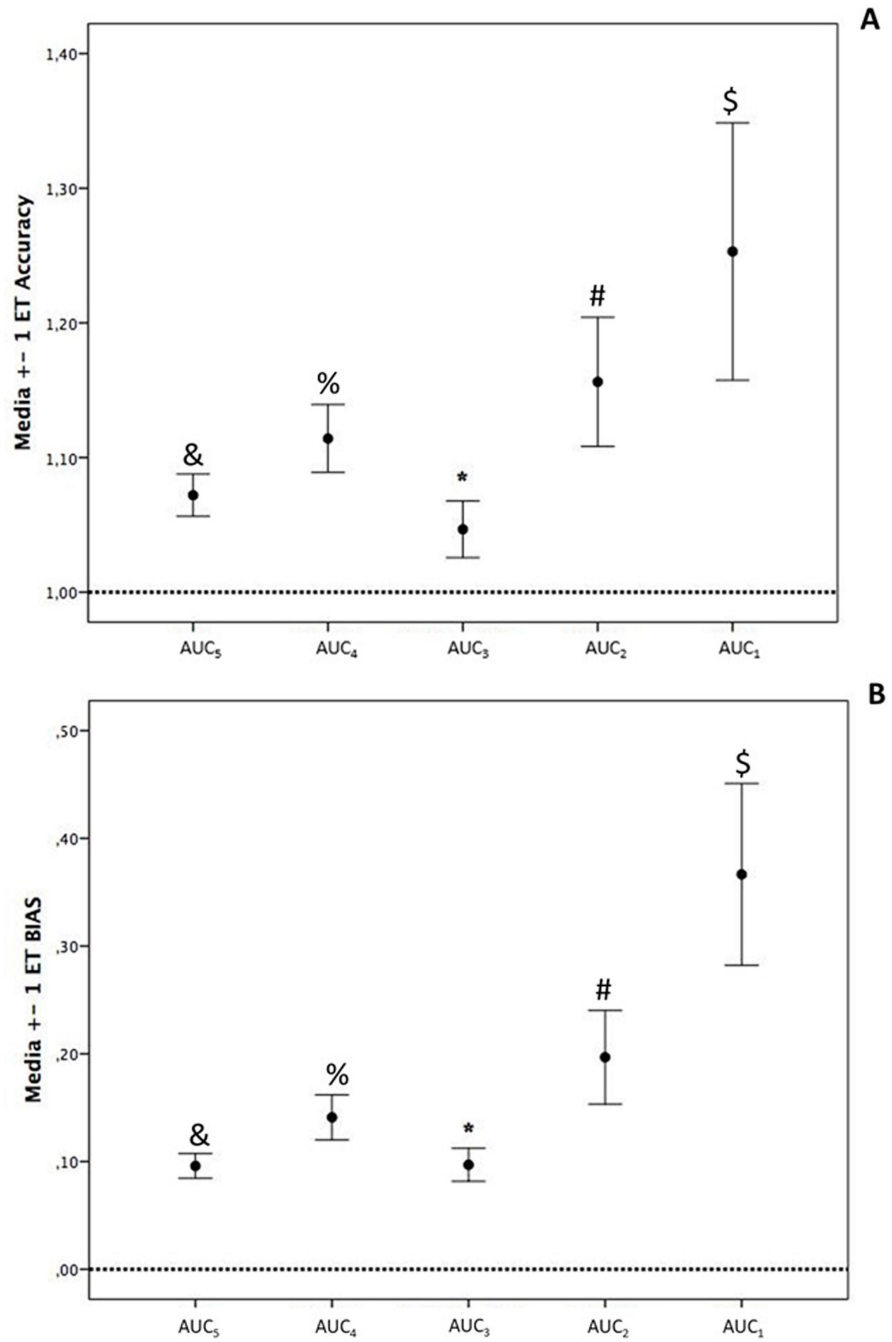
**(A)** Accuracy, where ^&^*p* < 0.01 vs. AUC-2, and *p* < 0.05 vs. AUC-4; ^%^*p* < 0.01 vs. AUC-1, and AUC-3; ^*^p < 0.01 vs. AUC-4, and *p* < 0.05 vs. AUC-5; ^#^*p* < 0.01 vs. AUC-3, and *p* < 0.05 vs. AUC-1; ^$^*p* < 0.05 vs. AUC-3. **(B)** Bias, where ^&^*p* < 0.01 vs. AUC-2, AUC-4, AUC-5, and *p* < 0.05 vs. AUC-3; ^%^*p* < 0.01 vs. AUC-1, and *p* < 0.05 vs. AUC-3, AUC-4, and AUC-5; ^*^p < 0.001 vs. AUC-5, and *p* < 0.01 vs. AUC-4, and *p* < 0.05 vs. AUC-1, and AUC 2; ^#^*p* < 0.01 vs. AUC-1, AUC-3, AUC-5, and *p* < 0.05 vs. AUC-2; ^$^*p* < 0.001 vs. AUC-1, AUC-2, AUC-3, and AUC-4.

In the correlation analysis, AUC_Ref_ showed the strongest correlation with AUC-1, AUC-2, and AUC-3, a moderate correlation with AUC-4, and a weak correlation with AUC-5 (Figure 4). Bland–Altman analysis (Figure 5) revealed that AUC-3 had the narrowest limits of agreement (LOA, 131) compared with AUC-1 (150), AUC-2 (184), AUC-4 (284), and AUC-5 (617). Furthermore, AUC-3 exhibited the smallest mean difference (−16) when compared with AUC-1 (−28), AUC-2 (−40), AUC-4 (−46), and AUC-5 (−60) (see Figure 6).

### 3.2 Pharmacokinetic and pharmacodynamic analysis

The AUC/MIC in 17 patients (47.2%) achieved subtherapeutic AUC/MIC values whereas 24 patients (66.7%) presented trough levels below 15 mg/dL. Another relevant finding was 13.9% supratherapeutic AUC/MIC values in contrast 5.6% supratherapeutic trough levels over 15 mg/dL (Table 5).

**Table 5:** Vancomycin AUC/MIC therapeutic values and vancomycin therapeutic trough levels, where AUC_24_ is area under the curve in 24 h; MIC is minimum inhibitory concentration.

Variable		Values, *N* (%)
AUC_24_/MIC, mg*h/L	Subtherapeutic	17 (47.2)
	Therapeutic	14 (38.8)
	Supratherapeutic	5 (13.9)
Trough level, mg/dL	Subtherapeutic	24 (66.7)
	Therapeutic	10 (27.8)
	Supratherapeutic	2 (5.6)

## 4 Discussion

In critically ill patients, our study underscores the significant challenge of achieving precise vancomycin dosing. It makes a valuable contribution to the field of vancomycin monitoring by emphasizing the potential for improved techniques and the need for continued advancements in this area. Through the comparison of five different strategies, our findings strongly support the use of at least two plasma levels to achieve greater therapeutic accuracy (31).

In agreement with previous studies, Flannery et al. (32) and AbuSara et al. (33), emphasize the importance of shifting from trough-based vancomycin monitoring to AUC-based dosing, as recommended by the 2020 IDSA-ASHP guidelines (1). Both studies (32, 33) highlight that trough levels (15–20 mg/L) do not reliably predict adequate AUC values, leading to potential subtherapeutic exposure or nephrotoxicity. Furthermore, consistent with AbuSara et al. (33) levels below 15 mg/L did not necessarily indicate subtherapeutic AUCs, and over half of patients with troughs in the 15–20 mg/L range had excessive AUC/MIC (>600 h^−1^), increasing nephrotoxicity risk.

Our study supports the evolving trend of emphasizing the AUC/MIC ratio over traditional trough level monitoring, aligning with recent clinical guidelines (1, 6). The observed discrepancies between reference and calculated AUC values in Bayesian-based monitoring reflect findings by Sujita et al. (25), who emphasize the need for further refinement of these algorithms in critically ill populations. While Turner et al. (26) highlighted the efficacy of PrecisePK^™^, our findings suggest that the number of plasma levels used plays a pivotal role in AUC accuracy. Specifically, relying solely on the trough level introduces significant variability in AUC estimation. In contrast, incorporating two plasma levels (e.g., peak and trough) improves accuracy and reduces bias, providing a practical and precise alternative for therapeutic monitoring.

The complexity of obtaining an accurate AUC/MIC ratio lies in the pharmacokinetic modeling of vancomycin’s bicompartmental distribution. Despite this complexity, our study demonstrates that Bayesian software can reduce the need for frequent plasma sampling without compromising the accuracy of AUC/MIC estimation (25).

An important observation in our study was that AUC_Ref_ values calculated using the trapezoidal rule were consistently lower than those estimated using the Bayesian approach. To minimize potential bias inherent to the trapezoidal method, vancomycin sampling times were selected to represent key pharmacokinetic phases (distribution, peak, and elimination) ensuring a more accurate estimation of the area under the curve. For this reason, a minimum of three plasma concentrations was required per patient; this strategy aimed to reduce variability and improve the reliability of the AUC_Ref_ calculation. Despite this, the trapezoidal method assumes linear interpolation between measured concentrations and may still underestimate true exposure, particularly when inflection points are missed (34). In contrast, Bayesian software tools generate full predicted concentration-time profiles using prior population models and individual patient data, which often leads to higher AUC estimates. It is also important to consider that Bayesian programs are inherently designed to prioritize patient safety. Consequently, when faced with uncertain or limited data, they may default to conservative assumptions, often resulting in higher AUC projections to reduce the risk of underdosing and potential treatment failure (10, 35). These differences highlight the strengths and limitations of both methods and underscore the need for clinical context when interpreting AUC values.

The observed discrepancy between subtherapeutic trough concentrations and adequate AUC/MIC ratios in some patients may be explained by individual pharmacokinetic variability, particularly in the critically ill. Several physiological factors—such as changes in renal clearance and volume of distribution—can significantly influence vancomycin exposure. For example, patients with preserved or augmented renal clearance (ARC) may exhibit rapid drug elimination, leading to lower trough levels, while still achieving an adequate AUC (36, 37). Additionally, an increased volume of distribution, frequently observed in ICU patients due to capillary leak, systemic inflammation, or aggressive fluid resuscitation, may contribute to lower plasma concentrations without necessarily reflecting subtherapeutic exposure (38). These findings highlight the limitations of trough-based monitoring and reinforce the value of AUC-guided dosing as a more reliable strategy for assessing therapeutic exposure and optimizing vancomycin therapy.

This study has several limitations. It was conducted in a single center with a limited sample size, which may affect the generalizability of the findings. The relatively small sample size and underrepresentation of females may impact the generalizability of our findings. Additionally, the relatively low incidence of renal failure in our cohort may limit the applicability of our findings to patients with significant organ dysfunction. Variability in renal function, a hallmark of critically ill patients, may have also influenced the pharmacokinetic modeling outcomes. While our results support the clinical utility of Bayesian-guided vancomycin dosing, further validation in larger, multicenter cohorts is necessary to confirm its reproducibility across diverse ICU populations. Future prospective studies should assess not only pharmacokinetic performance but also clinical outcomes, toxicity, and cost-effectiveness. Future research should involve larger, more diverse patient populations and focus on optimizing Bayesian algorithms to improve accuracy in this population.

Despite these limitations, our study has notable strengths. The 24/7 availability of vancomycin plasma level monitoring at our center enabled timely and accurate dosing adjustments. In fact, this allows achieving 108 blood samples for determination of vancomycin concentrations. The expertise of our ICU team in therapy administration and sample collection, coupled with the use of validated Bayesian pharmacokinetic software, enhanced the robustness of our findings. Furthermore, our focus on achieving PK/PD-aligned therapy in critically ill patients provides valuable insights into optimizing antibiotic treatment in intensive care settings.

In conclusion, this study suggests that obtaining at least two plasma levels is essential for optimal vancomycin monitoring in critically ill patients. These findings highlight the potential for improved accuracy and reduced bias in AUC estimation compared with single-level monitoring. Future studies should evaluate whether this approach leads to safer administration or improved clinical outcomes, paving the way for further advancements in vancomycin dosing strategies.

## Data Availability

The datasets presented in this article are not readily available because the datasets generated and/or analyzed during the current study are not publicly available due to ethical restrictions concerning patient privacy and confidentiality, but are available from the corresponding author on reasonable request. Requests to access the datasets should be directed to rene.lopezh@gmail.com.
